# Novel Approaches to Studying SLC13A5 Disease

**DOI:** 10.3390/metabo14020084

**Published:** 2024-01-24

**Authors:** Adriana S. Beltran

**Affiliations:** Department of Genetics, University of North Carolina, Chapel Hill, NC 27599, USA; beltran@med.unc.edu; Tel.: +1-(919)-537-9336

**Keywords:** SLC13A5, NaCT, iPSCs, neurons, hepatocytes, organoids

## Abstract

The role of the sodium citrate transporter (NaCT) SLC13A5 is multifaceted and context-dependent. While aberrant dysfunction leads to neonatal epilepsy, its therapeutic inhibition protects against metabolic disease. Notably, insights regarding the cellular and molecular mechanisms underlying these phenomena are limited due to the intricacy and complexity of the latent human physiology, which is poorly captured by existing animal models. This review explores innovative technologies aimed at bridging such a knowledge gap. First, I provide an overview of *SLC13A5* variants in the context of human disease and the specific cell types where the expression of the transporter has been observed. Next, I discuss current technologies for generating patient-specific induced pluripotent stem cells (iPSCs) and their inherent advantages and limitations, followed by a summary of the methods for differentiating iPSCs into neurons, hepatocytes, and organoids. Finally, I explore the relevance of these cellular models as platforms for delving into the intricate molecular and cellular mechanisms underlying SLC13A5-related disorders.

## 1. Introduction

SLC13A5 (Solute carrier family 13 member 5) deficiency causes neonatal epilepsy that is refractory to treatment [1,2]. This gene encodes a sodium-citrate transporter (NaCT) that belongs to the sodium dicarboxylate/sulfate cotransporter family [3]. NaCT is a protein found at the membrane of cells, and it is responsible for transporting citrate from the extracellular environment into the cell, where it plays a pivotal role in maintaining cellular metabolic homeostasis [4,5,6,7,8]. Pathogenic bi-allelic variants in the gene result in the loss of protein activity, affecting children within a few hours of life with frequent seizures [2]. Children with the same genetic variant present different behaviors and clinical responses, even in closely related genetic backgrounds [9]. Although the precise pathophysiology underlying how loss-of-function variants lead to the clinical phenotype remains unknown, the current hypothesis suggests that the depletion of intracellular citrate may disrupt cellular metabolism in neurons [10]. Cytoplasmic citrate is a crucial carbon source for neurotransmitters such as glutamate and GABA (gamma-aminobutyric acid) [11]. Therefore, the depletion of citrate within the cytoplasm may hinder the synthesis of GABA, potentially contributing to neurodevelopmental deficiencies and epileptic traits [12]. Conversely, inhibition of NaCT activity in the liver protects against metabolic diseases, including non-alcoholic fatty liver disease, obesity, and insulin resistance [13,14,15,16,17]. The biological and clinical relevance of the interaction between these disjoint mechanisms has not been actively studied. Consequently, this gap in knowledge and its impact on the clinical setting need to be clarified.

A challenge in studying SLC13A5 disease, especially epilepsy, is the limitation of available experimental models that accurately capture the complexity of the human brain. While mouse models offer versatility, they differ from human brains in their cellular and molecular composition [18,19]. In contrast, induced pluripotent stem cells (iPSCs) offer a promising alternative as they have the remarkable ability to differentiate into any cell type found in the human body, such as neurons and hepatocytes [20,21,22]. Furthermore, the iPSC-derived progeny exhibits significant phenotypic and morphological differences between disease-patient-derived cells and their healthy counterparts, better portraying human disease [23,24]. In the context of SLC13A5 disease, iPSCs provide a versatile platform for exploring the impact of *SLC13A5* variants in neurons and hepatocytes. In particular, iPSCs can define the potential connection between hepatocytes and neurons deficient in SLC13A5.

In this review, I provide an overview of the *SLC13A5* variants in the context of human disease and the specific cell types in which expression of the transporter has been observed. I describe current technologies to generate patient-specific iPSCs and summarize the methods for differentiating iPSCs into neurons, hepatocytes, and organoids while also discussing their inherent advantages and limitations. Finally, I discuss the relevance of these cellular systems as a platform for exploring the intricate molecular and cellular mechanisms underlying SLC13A5-related disorders. 

## 2. *SLC13A5* Variants

In humans, the presence of pathogenic bi-allelic variants in the *SLC13A5* gene is responsible for causing neonatal epilepsy, significant progressive cognitive and behavioral impairments, and poor tooth development [1,10,12,25,26]. Epileptic disorder is characterized by abnormal and excessive rhythmic brain activity, leading to spontaneous recurrent seizures. It appears to be a monogenic disorder, where epilepsy results from the malfunction of the NaCT. Notably, all tested mutations in this gene show reduced quantities or incorrect localization of the transporter within cells [1,2,27]. In line with these findings, patients suffering from this condition exhibit elevated levels of citrate in their cerebrospinal fluid and blood [10]. The epileptic episodes manifest as convulsive events with different types of seizures, including hemiclonic, myoclonic, and generalized tonic-clonic seizures. Also, some patients experience subclinical seizures, nonconvulsive status epilepticus, and absence or atypical absence seizures [1,2,28]. The primary treatment for these seizures involves anti-seizure medications, although their effectiveness in controlling seizures varies among affected children [9,29]. Despite identifying the pathogenic variants, our understanding of the molecular mechanisms that drive the development of neuronal dysfunction and epilepsy in this context is minimal. This knowledge gap poses a significant challenge, impeding the development of much-needed targeted therapeutic strategies for SLC13A5 deficiency patients. 

**Figure 1:**
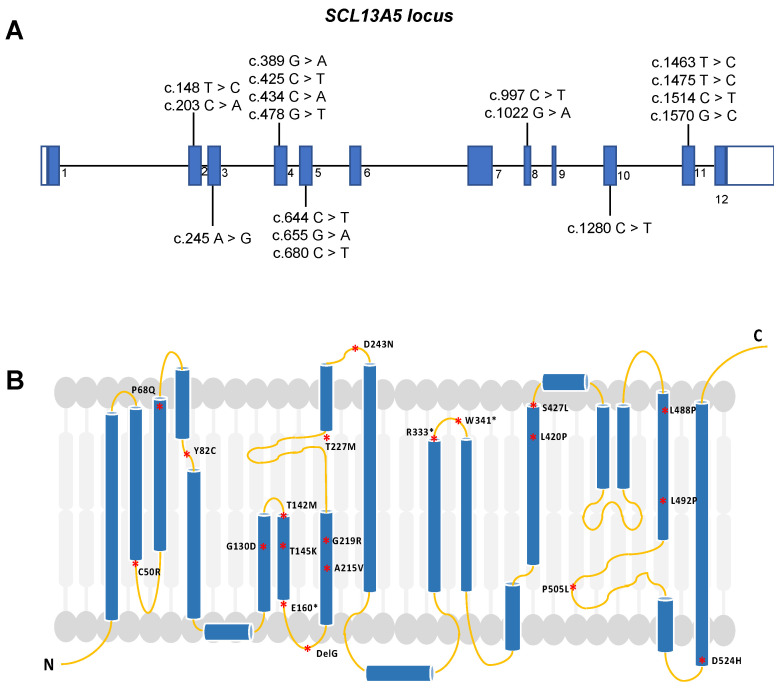
Diagram illustrating potential disease-causing mutations in SLC13A5. (**A**) *SLC13A5* gene locus with exons sequentially numbered from 1 to 12. Here, the variants are shown at their specific locations within the exons, designated as c.148T > C; “c.” denotes the coding DNA sequence, “148” specifies the nucleotide position, and “T > C” describes the nucleotide substitution (a thymine replaced by cytosine). (**B**) SLC13A5 protein model displaying the resultant amino acid substitutions in the protein caused by the variants described in A. The amino acid changes in the protein are denoted by an asterisk (*), with the “P68Q” signifying a change from the amino acid proline (P) to glutamine (Q). The ‘N’ refers to the free amine end, known as the ‘N-terminus’, while ‘C’ denotes the carboxyl terminus, referred to as the ‘C-terminus’, of the protein. The image in (**B**) was adapted from [30,33,34].

The human *SLC13A5* gene is located on chromosome 17, has a length of 30 kb, and contains 12 exons [4]. Pathogenic variants in any of the exons can directly affect protein expression, structure, and activity (Figure 1). So far, more than forty potentially pathogenic variants have been identified, with some altering the primary and secondary structures of the protein [25,30,31]. Functional transport studies conducted in vitro have revealed that these variants lead to minimal or no transport activity, suggesting a prevailing loss-of-function mechanism [27,32,33]. These variants potentially change the intrinsic citrate uptake function, significantly impacting children with seizures shortly after birth. A noteworthy aspect is that individuals harboring the same *SLC13A5* variants can exhibit varying frequencies of seizures and degrees of developmental disability. This observation suggests that *SLC13A5* variant-induced deficiency is a heterogeneous disease with a broad spectrum of clinical manifestations. 

SLC13A5 deficiency diagnosis is performed by whole exome sequencing (WES) or by a targeted sequencing panel (SLC13A5 is included in multiple epilepsy panels) on affected patients [10]. To date, more than 95 patients have been diagnosed with epilepsy, harboring a compound heterozygous mutation of those 40 variants [25,30,33,34]. These variants primarily affect the first or second sodium binding sites, suggesting that mutations may disrupt citrate uptake, potentially affecting the biological functions of the NaCT protein (Figure 1) [29,33]. Using a homology model approach, *SLC13A5* variants are classified based on their likely impact on transport function and protein expression, trafficking, and stability [33]. For example, the T142M mutation directly affects sodium ions (Na+) or citrate binding, while C50R, H106R, and G417E, which are located at the interface of the scaffold and transport pore, may perturb the sliding of the transport domain up and down the scaffold domain [33].

Missense mutations, on the other hand, lead to protein-folding and trafficking defects, resulting in misfolded proteins that fail to pass quality control and cannot exit the endoplasmic reticulum to reach the Golgi apparatus for mature glycosylation. Examples of mutations in the scaffold or transport domain include C50R, P68Q, Y82C, L111R, G417E, G423E, S427L, G130D, T145K, G219R, P487L, L488P, L492P, and D524H [33]. Notably, G219R is the most common disease-causing mutation, and its location near the interhelix loop disrupts local structure due to steric clashes with nearby residues, affecting the entire protein and leading to premature degradation in the endoplasmic reticulum [33]. 

Other types of mutations include nonsense mutations or stop codons that interfere with protein synthesis, splicing mutations, and promoter variants that may still display residual activity [27,32,33]. It is essential to note that there is one *SLC13A5* variant with gain-of-function that increases citrate transport [3,33]. Variants can have different effects on citrate transport rates, and their impact on substrate specificity may differ under nutrient-limited conditions [7]. Whether these *SLC13A5* variants indeed alter protein levels that contribute to the neurological phenotype or lead to other non-neurological consequences remains largely unknown 

## 3. SLC13A5 Expression in Different Cell Types

Expression of *SLC13A5* mRNA varies across tissues and species. Although in other mouse species the expression is higher in the brain, in humans, the liver shows the highest mRNA levels compared to the brain, spleen, and testis [15,32]. Also, in the liver, there is well-documented evidence that NaCT is expressed at substantial levels in hepatocytes, where it localizes to the sinusoidal membrane, interfacing with the bloodstream [13]. Notably, there is a lack of published reports on the NaCT protein expression in human neurons or brain tissue, despite the importance of SLC13A5 in epilepsy. Thus, in the context of human pathology, understanding the expression of SLC13A5 in the liver and brain is particularly relevant.

In animals, *SLC13A5* mRNA is found in neurons of the cerebral cortex, cerebellum, and olfactory bulb [35,36]. Expression in the brain increases during postnatal development and stabilizes in adult animals. It is present in human astrocytes and mouse cerebrocortical astrocytes but absent in rat astrocytes, where it is expressed in neurons cultured in vitro [35]. In the testis, it is exclusively expressed in germ cells. These distinct expression patterns across different cell types suggest that SLC13A5 has diverse functions and potentially plays crucial roles in various physiological processes, including cellular metabolism and neuronal function. 

### 3.1. SLC13A5 Expression and Function in the Brain

*Slc13a5* mRNA expression in the mouse brain has been extensively documented. Within the mouse cerebral cortex, this expression appears to be predominantly limited to neurons in specific regions such as the cerebral cortex itself, the hippocampal formation, the cerebellum, and the olfactory bulb [5]. Interestingly, NaCT deficiency in the brain leads to a more severe disease despite the unknown role of neuronal excitability or functionality [12,37]. 

Mice with *Slc13a5* gene deletion exhibit altered extracellular and intracellular citrate levels in the brain [38]. Importantly, in vitro, cells overexpressing either the wildtype or mutant transporter display the same levels of endogenous citrate [2]. However, it remains unclear how these alterations in citrate levels lead to epilepsy in humans. Current hypotheses from animal models suggest SLC13A5 deficiency leads to systemic metabolic dysregulation [39,40] and alterations in citrate levels that result in neuronal network excitability and increased seizure propensity [38]. Mice with neuron-specific overexpression of *Slc13a5* display autistic-like behaviors linked to changes in citrate and coenzyme A (CoA) metabolism [40]. 

Furthermore, wildtype NaCT can import exogenous citrate, which is then utilized in the metabolism of fatty acids and tricarboxylic acid (TCA) cycle intermediates. This metabolic activity, occurring under hypoxic conditions with reduced pyruvate dehydrogenase flux and limited glutamine (and other nutrients) availability, can catabolize citrate in the cytosol to support acetyl-CoA generation [7,40]. Interestingly, patients display abnormalities in fatty acid synthesis and energy generation [39]. Although some of these patients have greatly benefited from ketogenic diets that shift the utilization of carbohydrates to fat for metabolic purposes [2], other patients exhibit worse symptoms [27]. Thus, it is plausible that abnormalities in the regulation of the metabolic component induce seizures.

Given the SLC13A5 expression in neurons, transient expression of SLC13A5 may be relevant for studies involving neuronal differentiation. Modulating citrate transport during neural differentiation could impact cellular processes and potentially influence cell fate decisions. For example, neural stem cells respond to succinate stimulation by upregulating Slc13a5, a response that depends on *Sucnr1* (Succinate receptor 1) signaling [41]. Slc13a5 is also involved in co-transporting succinate [42]. In vitro experiments with neural stem cells exposed to succinate have shown an increase in the expression of Slc13a5 and enhanced uptake of extracellular succinate. Furthermore, in vivo experiments have shown that Slc13a5 effectively removes extracellular local succinate when neural stem cells are introduced into experimental autoimmune encephalomyelitis mice via cerebrospinal fluid circulation [41]. This data aligns with current hypotheses suggesting that Slc13a5 deficiency leads to metabolic dysregulation, resulting in alterations in citrate levels. These changes in citrate could contribute to increased neuronal network excitability and a higher propensity for seizures.

Additionally, Slc13a5 has been shown to play a role in protecting cells from high zinc concentrations. Slc13a5 knockout cells exhibit reduced viability in response to elevated zinc levels, indicating that NaCT function is necessary for protection against zinc toxicity in high citrate environments. In this context, Slc13a5-mutant neurons may be susceptible to increased synaptic function due to variations in zinc concentration during neuronal activation [12]. Improper NaCT function has also been linked to elevated synaptic citrate concentrations, which enhance synaptic methyl-D-aspartate (NMDA) receptor function and cause neuronal dysfunction [12]. Further studies in a human cell system are required to investigate the downstream effects of NaCT deficiency in detail and explore the involvement of neuronal energy supply, neurotransmitter production, and modification of neuronal activity as potential pathogenic mechanisms in Slc13a5 deficiency.

### 3.2. SLC13A5 Expression and Function in the Liver

The *SLC13A5* gene exhibits higher expression levels in hepatocytes, where the NaCT localizes to the sinusoidal membrane in direct contact with the bloodstream [13]. This transporter contributes to maintaining a stable level of citrate in circulation, typically ranging from 50 to 150 μM, and serves as a backup energy source for metabolic processes. Consequently, intracellular citrate levels within hepatocytes are meticulously regulated through a delicate balance between biosynthesis and transport. Cytosolic citrate is a critical precursor and regulator of de novo fatty acid synthesis. This complex regulation makes citrate a key metabolite intricately linked to hepatic glucose and fatty acid metabolism [7]. 

Recent studies have indicated that the increased activity of NaCT contributes to certain conditions, such as obesity, non-alcoholic fatty liver disease (NAFLD), high-fat diet treatment in rhesus monkeys, and exposure to xenobiotics in human and rat hepatocytes [13,14,15,16,17,43]. This suggests that NaCT activity may serve as a risk factor for metabolic disorders. Functional studies have proposed that inhibiting or knocking down NaCT in hepatocytes can impact cellular processes systemically, which may not necessarily depend on the presence of extracellular citrate [44]. 

Furthermore, the regulation of the *SLC13A5* gene appears to be sensitive to different mechanisms. For instance, phenobarbital has been shown to elevate *SLC13A5* mRNA and protein expression through a pregnane X receptor (PXR)-dependent signaling pathway in human primary hepatocytes, independently of constitutive androstane receptor signaling [43]. Conversely, genetic knockdown or pharmacological inhibition of PXR significantly attenuated this induction. Additionally, SLC13A5 is subject to regulation by glucagon and insulin in the liver, aiding in the regulation of blood glucose levels by promoting citrate uptake and its subsequent conversion to fatty acids [17]. The intake of dietary citrate can also impact citrate levels, although not SLC13A5 activity, as citrate uptake in the gut and kidneys is mediated by SLC13A2 [44].

Moreover, the deletion of Slc13a5 has been shown to have protective effects against high-fat diet-induced insulin resistance and has attenuated hepatic gluconeogenesis and lipogenesis [15,45]. Patients with variants in *SLC13A5* exhibit increased citrate levels in their body fluids [46]. Whether this is the result of deficient liver uptake remains currently unknown, emphasizing the need for further research in this area.

### 3.3. SLC13A5 Expression and Function in Various Cell Types

In addition to hepatocytes and neurons, *SLC13A5* has been detected in several other cell types, often in a transient manner. For instance, the transient expression of SLC13A5 can modulate the availability of citrate to alter cellular energy production and metabolic pathways, which are particularly relevant when cells are transitioning between different metabolic states, such as during cellular differentiation.

Transiently expressing *SLC13A5* in undifferentiated cells, for example, can impact their metabolic status and facilitate their transition into a differentiated state. One illustrative example is observed in undifferentiated and early-stage differentiating mesenchymal stem cells, where increased SLC13A5 levels facilitate the import of citrate, boosting cellular energy levels crucial for osteophenotype progression during bone formation. This metabolic shift from glycolysis to oxidative respiration in response to osteostimulation generates more ATP, supporting the production of abundant matrix proteins required for high energy demands [47]. Consequently, the loss of function can impact osteogenic differentiation [48]. Importantly, mouse models and children with SLC13A5 deficiency display tooth decay and defective enamel [2,26]. Therefore, understanding how the transient expression of *SLC13A5* in mesenchymal stem cells during tooth development contributes to the development and function of teeth and bones is of great significance in the context of children with SLC13A5 deficiency.

This phenomenon of transiently expressing SLC13A5 to support cellular transitions between different cell fates can also be observed in Drosophila. In Drosophila, citrate plays a critical role in the communication and maturation of sperm. Cellular communication is mediated by the close spatial organization of the fly’s testis and midgut. The JAK/STAT signaling pathway is activated in the R4 (region 4) midgut region, leading to increased expression of intestinal sugar genes and the production of cytosolic citrate. Slc13a5 transports citrate from the R4 midgut region to the testis, promoting sperm maturation. Reduced citrate efflux negatively affects sperm maturation and decreases spermatocyte numbers due to metabolic changes in the testis. In males, citrate serves as a signal that regulates sex-specific differences in sugar gene expression and increases food intake [49]. Recent data have even shown that the TCA cycle is dispensable for sperm differentiation in flies and that the germline depends on external citrate import for differentiation [50]. This work suggests that circulating citrate can have systemic effects outside of the producing cells. Therefore, the regulated expression of SLC13A5 is critical for maintaining metabolic homeostasis across cell types. 

## 4. Physiologically Relevant Cellular Models to Study Human SLC13A5 Disease

**Figure 2:**
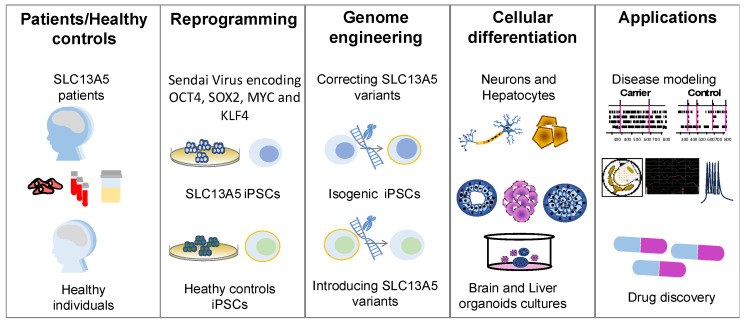
Research workflow using induced pluripotent stem cell (iPSCs) derived cellular models. Illustration of the research procedures for iPSC disease modeling and therapeutic investigations, incorporating both patient-derived and healthy control samples. The process begins with the collection of somatic cells, such as skin fibroblast, blood, or urine cells, from either a patient or a healthy individual, followed by the reprogramming of these samples into iPSCs. Genetic editing techniques such as clustered regularly interspaced short palindromic repeats (CRISPR) may be utilized to create isogenic controls. This involves either correcting pathogenic variants in patient-derived iPSCs or introducing such variants into iPSC-derived from healthy individuals. Next, these iPSCs can be guided to differentiate into disease-relevant cell types, such as neurons and hepatocytes, using either 2D or 3D differentiation protocols. Multiple in vitro assays are then applied to these cell types, aiming to replicate disease phenotypes across functional, morphological, and biochemical aspects. Once a robust and reproducible in vitro disease phenotype is established, the drug discovery phase can commence.

A significant challenge in SLC13A5 epilepsy research is the absence of experimental models capable of faithfully replicating the complexity of human physiology, especially the complexities of neuronal circuits, while remaining accessible and easily manipulatable. While intact in vivo animal models, particularly mice, offer versatility for studying gene defects and circuit abnormalities, they come with their limitations. Notably, murine brain development and the associated cellular and molecular composition differ from those of humans [51]. IPSCs have emerged as valuable tools for creating various patient-specific models. In the context of SLC13A5 disease, iPSCs offer a versatile platform for investigating the effects of variants in cell types relevant to the disease (Figure 2). The following sections provide an overview of the current technologies used to generate patient-specific iPSCs and differentiate them into neurons, hepatocytes, and organoids.

### 4.1. Induced Pluripotent Stem Cells Models

Induced pluripotent stem cells (iPSCs) play a pivotal role in disease modeling, drug discovery, and regenerative medicine [52,53,54]. Unlike embryonic stem cells (ESCs), which are cells derived from the inner cell mass of embryos, iPSCs are derived from somatic cells forced to a pluripotent state [21,22,55,56]. IPSCs share similarities with ESCs in terms of their cellular characteristics, self-renewal capacity, and ability to differentiate into virtually any cell type [20,57,58]. Remarkably, these similarities persist despite reports of molecular heterogeneity associated with epigenetic variations, often attributed to individual cellular differences and the erasure of epigenetic memory in iPSCs [59,60]. 

The unique feature of iPSCs is their ability to generate patient-specific or phenotypic-specific allogeneic models to study certain diseases or for use in cellular therapy. This approach minimizes the risk of immune rejection and host versus graft disease [61,62]. As a result, iPSCs pave the way for personalized medicine, where treatment strategies can be tailored to a patient’s genetic background, potentially enhancing the effectiveness of interventions while reducing adverse reactions.

**Figure 3:**
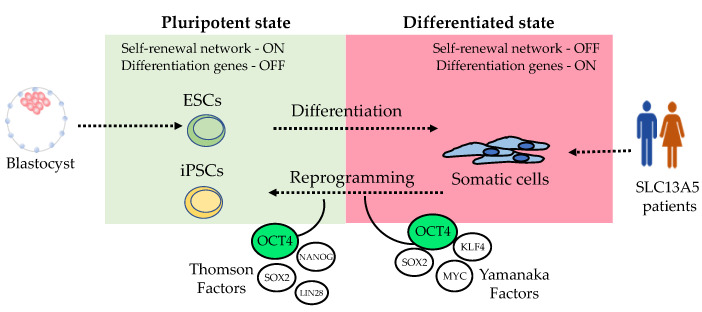
Maintenance and induction of pluripotency. This figure illustrates the regulation of stem cell-like properties, often referred to as the “stemness” phenotype. In embryonic stem cells (ESCs), the network of transcription factors that maintain stemness is active, while the transcription factors that drive differentiation are inactive. By artificially activating the stemness transcription factor network in mature, specialized cells, we can induce a state of pluripotency, creating induced pluripotent stem cells (iPSCs). These iPSCs exhibit characteristics similar to those of ESCs. The transition to this pluripotent state is a dynamic process that can be triggered by various combinations of transcription factors. Notable examples include the Yamanaka factors (OCT4, SOX2, KLF4, and MYC) [20] and the Thomson factors (OCT4, SOX2, NANOG, and LIN28) [21].

Embryonic stem cells maintain their pluripotency through a signaling network involving the transcription factors Octamer-binding transcription factor 4 (*POU5F1*, OCT3/4), SRY-Box Transcription Factor 2 (*SOX2*), and Nanog Homeobox (*NANOG*) [53,54]. This signaling network maintains pluripotency by promoting gene expression of genes controlling self-renewal and pluripotency while simultaneously inhibiting genes associated with the process of differentiation [52]. Similarly, artificially inducing the expression of those transcription factors in somatic cells leads to a reprogrammed or “embryonic like” cellular phenotype (Figure 3). Various transcription factors involved in maintaining stemness in ESCs have shown the capacity to reprogram somatic cells with similar efficiency. The pioneering work of Shinya Yamanaka led to the discovery of the first specific combination of transcription factors capable of inducing stemness and reprogramming somatic cells into iPSCs [20]. Yamanaka used lentiviral overexpression of *OCT4*, *SOX2*, *KLF4* (Kruppel Like Factor 4), and *MYC* (MYC proto-oncogene) to induce dermal fibroblast to form colonies morphologically resembling embryonic ones. This reprogramming phenomenon was subsequently replicated by James Thomson, albeit with a slightly different set of transcription factors, *OCT4* and *SOX2*, alongside *NANOG* and *LIN28* [21]. Remarkably, these experiments confirmed the plasticity of the reprogramming process, which we continue to explore to this day.

The pluripotency and differentiation capabilities of iPSCs were later demonstrated by directly differentiating them into neural and cardiac cells using a spontaneous differentiation method [21,63]. Over the years, various sets of reprogramming transcription factors have been employed. All reprogramming sets have *OCT4*, which remains the “conserved” or “indispensable” factor for reprogramming somatic cells into a pluripotent state (Figure 3). This is likely because *OCT4* plays a central role as the master regulator of the stemness signaling network [52,53,54]. Typically, the reprogramming “cocktail” includes transcription factors that promote stemness, such as *OCT4* and *NANOG*; factors related to cell growth, such as *MYC*; and factors that support cell survival during cellular fate changes, such as *KLF4* [52,64,65].

Human iPSCs can be derived from theoretically any cell type [56,66]. While fibroblast, blood cells, and urine cells are commonly used in reprogramming experiments, blood cells are particularly favored due to their minimally invasive collection and being readily available from patient samples. Moreover, blood samples are often stored in biobanks, enabling retrospective studies [66,67]. The reprogramming methods have evolved over time as our understanding of how stemness transcription factors maintain pluripotency has expanded. Currently, all methods involve inducing the expression of these stemness transcription factors, whether by encoding them in retroviruses, lentiviruses, cytoplasmic viruses, episomal plasmids, the mRNA of the transcription factors, or recombinant proteins [68,69,70,71]. While the goal is to reactivate the endogenous genes to induce a pluripotent state, the efficiency and downstream effects differ for each method. Improvements have been made by combining different stemness transcription factors and cell types. Newer methods aim to avoid inducing genome instability and seek to enhance reprogramming efficiency [67,72].

Successful non-integrating methods have been developed, including episomal plasmids encoding *OCT4*, *SOX2*, *KLF4*, *MYC*, *LIN28*, *EBNA1* (Epstein–Barr nuclear antigen-1) and optionally *NANOG* [68]. Additionally, shRNA targeting the tumor protein P53 (*TP53*) has been employed to enhance reprogramming efficiency [67]. These modifications have significantly improved the reprogramming process. In the context of human research and cell therapy, integration-free methods are preferred as they do not entail permanent genomic alterations, and the plasmids can be eliminated from cells in a few passages [69].

One of the most widely adopted reprogramming strategies currently involves an integration-free method utilizing an RNA virus called Sendai virus (SeV) or Murine parainfluenza virus. This method encodes the “Yamanaka factors” (*OCT4*, *SOX2*, *KLF4*, and *MYC*) [72]. It is particularly favored for clinical applications due to its non-integration into the host cell’s genome for replication, the ability to be fully removed after several passages, and its compliance with Good Manufacturing Practice (GMP) standards [71]. In addition to viral-based methods, small molecules have been employed to reprogram or transdifferentiate patient-derived human samples into various cell types directly [70,73]. These chemical alternatives are potentially safer since most chemicals degrade rapidly, although they may take a considerably longer time to reactivate the expression of stemness transcription factors [73].

The success of reprogramming can be verified through various tools that assess both morphological and functional traits. Morphological resemblance to ESCs is characterized by features such as large nuclei, compact colonies, and small cytoplasm. Functional studies involve the expression of stemness genes, the presence of protein surface markers, and the ability to differentiate into the three germ layers [58,74,75,76]. It is essential to assess genetic stability through cell karyotype [77,78], and confirm a fully reprogrammed phenotype by RNA sequencing and methylation analysis [79,80,81,82]. 

The ultimate goal, for both biological and clinical applications, is the differentiation of iPSCs into specific cell types. While many cell types can be derived from iPSCs through the optimization of growth factors, signaling molecules, and culture conditions, it is crucial to ensure consistent and efficient differentiation. However, it is important to note that current iPSC-derived progeny may not always fully resemble their natural counterparts in terms of maturity and functionality, though achieving functional equivalence with native cells is critical for therapeutic success. Therefore, significant efforts are dedicated to obtaining faithful replicas of human cells, ensuring their freedom from residual undifferentiated cells, and establishing stable, controlled growth characteristics, which are essential for safe clinical applications, drug discovery, and accurate disease modeling.

### 4.2. Human Neurons Derived from Human Pluripotent Stem Cells

Studying the human brain on a cellular and molecular scale presents significant challenges [83]. Thus, neurons derived from iPSCs offer a unique advantage for both research and potential therapeutic applications. IPSC-derived neurons from patients carrying genetic mutations provide a valuable tool for investigating disease mechanisms, screening potential drug candidates, and testing therapeutic interventions in a controlled in vitro environment [84,85,86]. 

The traditional methodology for differentiating human iPSCs into neurons follows a systematic procedure. It begins by guiding the iPSCs to form embryoid bodies (EBs), which then differentiate into neural progenitor cells in the form of neurospheres [87]. These neural progenitor cells subsequently mature into functional neurons. This approach combines both 3D and 2D environments and is based on neurodevelopmental studies in animal models, which have identified key stages in mammalian neural cell fate commitment. Importantly, this procedure closely replicates the developmental timeline of human neurons in vivo [51,88].

Neural induction in the early phases of embryonic development relies on the intricate interactions among various signaling pathways, including bone morphogenetic protein (BMP), fibroblast growth factor (FGF), transforming growth factor β (TGFβ), sonic hedgehog (SHH), Notch, WNT, and retinoic acid (RA) [89,90,91,92]. Modulating these pathways with small molecules in a temporal and dose-dependent manner can guide iPSCs toward differentiation into specific types of neural cells. The most commonly used method involves inhibiting the SMAD signaling pathways using small molecules to drive iPSC differentiation into neural cells [93]. Once neuronal fate is established, molecules like fibroblast growth factor 2 (*FGF2*) can be used to maintain and promote the proliferation of neuronal progenitor cells [94]. Additionally, small molecules such as RA can be employed to influence the regional identity of the neurons [95]. Higher concentrations of RA promote posterior identity, and lower levels promote anterior identity [95,96]. In some cases, neuronal cultures are treated with antimitotic components to inhibit the proliferation of non-mitotic cells and enable mitotic neurons to survive. Finally, verification of successful differentiation and maturity is assessed at each stage to ensure consistent production of the desired cell type from batch to batch. In addition to morphological features, key markers are utilized to confirm cell fate, while the functional maturity of neurons is evaluated by measures of synaptic function.

In addition to traditional neuronal differentiation techniques relying on small molecules, iPSCs can also generate functional neurons by expressing specific transcription factors. For instance, transcription factors like *BRN2*, *ASCL1*, and *MYT1L* can facilitate the conversion of iPSCs into glutamatergic neurons [97]. Similarly, the expression of *NEUROG2* or *NEUROD1* in iPSCs can yield highly pure neurons in under two weeks [98]. This methodology has gained popularity for generating diverse neuronal and glial cells due to its high efficiency in both differentiation and maturation, resulting in a uniform population of matured cells [98,99]. Overcoming the variability in the differentiation process can reduce heterogeneity and provide reproducible results, especially when iPSC-derived neurons are immature. This can be particularly valuable for modeling late-onset neurological diseases or studying age-related aspects of neuronal function. Methods like virus transduction are utilized to introduce the transcription factors, or they are engineered into a safe harbor locus, often in a system that can be induced [100,101]. However, a potential caveat is that with viruses incorporated into the iPSCs’ genome, there’s a chance that the overexpression of transcription factors might obscure subtle developmental characteristics. Moreover, it remains uncertain whether diseases can be entirely mimicked under these conditions. Nevertheless, this strategy allows for scalability, which is essential for high-throughput drug screening and large-scale studies.

**Figure 4:**
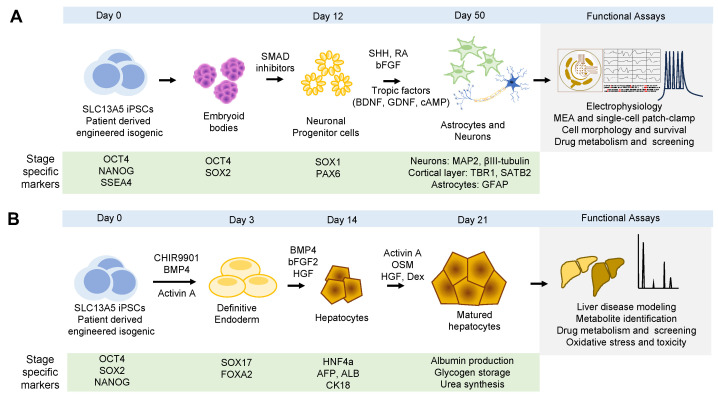
Diagram detailing the methods for differentiating induced pluripotent stem cells (iPSCs) into brain and liver cells. (**A**) Method to differentiate iPSCs into brain cells, including neurons and astrocytes, using a combination of small molecules and both 2D and 3D cultures. The process involves various stages where specific markers are used to confirm cell identity: OCT4 and NANOG for the loss of pluripotency; SRY-Box Transcription Factor 1 (SOX1) and paired box 6 (PAX6) for early neuronal progenitors; T-Brain-1 (TBR1) and Special AT-rich sequence-binding protein 2 (SATB2) for cortical layer neuronal progenitors. Pan-neuronal markers like microtubule-associated protein 2 (MAP2), βIII-tubulin, and synaptophysin, along with postsynaptic density 95 (PSD95), are used to evaluate the health and structure of neuronal networks. Glial fibrillary acidic protein (GFAP) is used to identify astrocytes. (**B**) Differentiation of iPSCs into hepatocytes. Sex-determining region Y-box 17 (SOX17) and forkhead box A2 (FOXA2) serve as markers for the definitive endoderm stage, while hepatocyte nuclear factor 4 alpha (HNF4a), alpha-fetoprotein (AFP), albumin (ALB), and cytokeratin 18 (CK18) indicate hepatic specification. Mature hepatocytes are identified by their expression of enzymes like tryptophan-oxygenase, tyrosine amino-transferase, and various cytochrome enzymes. The diagram shows the estimated timeline for these processes, from induction (Day 0) to mature neurons (Day 50) and hepatocytes (Day 21), and assays used to evaluate the iPSC-derived progeny functionality.

Validation of cell types and confirmation of cellular identity are achieved using specific antibodies against markers for each cell fate (Figure 4A). Early during the differentiation process, loss of pluripotency is assessed by the loss of expression of *OCT4* and *NANOG*. Early neuronal progenitors are often detected with *SOX1* (SRY-Box Transcription Factor 1) and *PAX6* (paired box 6), while late progenitors are detected with specific markers of each specific neuronal type. For example, for cortical layer-specific markers, *TBR1* (T-Brain-1), *CTIP2* (COUP-TF-interacting protein 2), *CUX1* (Cut-like homeobox 1), and *SATB2* (Special AT-rich sequence-binding protein 2) are used [102]. To assess neuronal network health, morphology, and composition, pan-neuronal markers such as *MAP2* (microtubule associated protein 2), βIII-tubulin (βIII-tubulin), *SYP* (synaptophysin), *PSD95* (postsynaptic density 95), *MUNC18* (mammalian uncoordinated-18), and *HOMER1* (Homer protein homolog 1), among others, can be employed [103].

Neuronal function of the iPSC-derived neurons can be assessed by measuring synaptic activity utilizing the classic single-cell patch clamp recordings or using new methodologies such as multielectrode array (MEA) plates [104]. Patch clamp recordings can measure action potentials in single iPSC-derived neurons, while MEA can assess the function of the entire neuronal network. MEA plates consist of an electrode grid that detects and records action potentials in individual neurons and tracks their propagation across the neuronal network. Daily recordings track network development and maturation, as well as neuronal activity, synchronicity, and oscillation [104,105,106]. All these parameters are important for determining the burst frequency of action potentials and synchronization of a neuronal population, which is characteristic of seizures when hyperactive. Long-term culture of iPSC-derived neurons allows the monitoring of disease progression over extended periods, which can be challenging with animal models or post-mortem human brain tissues.

MEAs technology has been used to assess excitatory neuron activity in iPSC-derived neurons affected by genetic diseases, such as deficiencies in voltage-gated potassium and sodium channels, including *KCNQ2*, *KCNT1*, and *SCN8A* [106,107,108,109]. MEA findings of real-time neuronal network activity on potassium channels align with earlier observations from mouse and cell culture models, confirming these channels play a critical role in regulating neuronal excitability and that their dysfunction or blockage leads to epileptogenic activity [109]. In principle, MEA could also track changes in neuronal excitability due to persistent sodium currents [105,106]. Sodium channels transiently allow sodium ions into the cell for action potential generation and propagation before the channel inactivates. When a small fraction of the sodium current persists even during prolonged depolarization, it causes a persistent sodium current that can lead to increased neuronal excitability [110]. This capability of MEA to detect and confirm changes in neuronal excitability and seizure activity due to malfunctioning voltage-gated channels is invaluable for epilepsy research [106,107,108].

One unique feature of MEA is its capacity to capture longitudinal data to evaluate the activity of patient-derived neurons and isogenic controls to recapitulate the patient’s epileptic phenotype. This involves evaluating measurements over time and detecting consistency, variations, and potential correlations or causations. MEA synthesizes data from weeks of recordings, identifying both simple and complex activity patterns at single-electrode and network levels. This includes spikes, bursts, and synchronized network events. Statistical analysis is incorporated to discern the significance of differences in genetic models and treatment responses, aiding in understanding disease progression and treatment effects [105,106,109]. Crucial for studying dynamic biological processes, MEA can detect seizure initiation, duration, and cessation in the context of SLC13A5 mutations. Despite the complexity and evolving nature of data analysis, integrating MEA with iPSC-derived neurons and combining it with patch clamp recording provides a comprehensive approach to understanding epilepsy mechanisms and developing targeted therapies.

### 4.3. Human Hepatocytes Induced from Induced Pluripotent Stem Cells

Similar to iPSC-derived neurons, the differentiation of iPSCs into hepatocytes involves the guidance of cells through the different stages of liver development. This is achieved by utilizing combinations of growth factors and small molecules to mimic the intrinsic signaling pathways during embryonic liver development [111]. Current techniques commonly incorporate specific combinations of small molecules to enhance the efficiency of differentiation. This enhancement is typically assessed by the expression of definitive endoderm and hepatocyte markers, as well as the demonstration of crucial liver functions like cytochrome P450 enzyme activity, glycogen synthesis, albumin secretion, and drug processing [111,112,113]. 

It is important to recognize the distinct spatial organization of liver cells within their native environment. In the liver lobules, progenitor cells are situated in the periportal regions, while mature hepatocytes are found in the pericentral region [114]. Consequently, iPSC-derived hepatocytes often exhibit variations in differentiation efficiency and function, frequently resembling fetal or neonatal hepatocytes rather than fully mature adult hepatocytes [111,112,115]. One promising strategy is the utilization of 3D culture methods, which offer a more comprehensive representation of various stages of liver development. To accurately represent liver organization, methods that closely mirror in vivo cellular environments are recommended, as they can improve cell–cell interactions, nutrient circulation, waste removal, and promote maturation [116]. Achieving complete maturation may necessitate specialized culture conditions, the inclusion of maturation factors, or extended culture durations to attain consistent and mature results [117]. 

To ensure successful hepatocyte differentiation, it is crucial to conduct a thorough evaluation at each stage of the differentiation process (Figure 4B). This evaluation encompasses the examination of cell morphology, the measurement of gene and protein marker expression levels, and the assessment of enzyme activity [118]. Markers and functional assays are used to distinguish stages of differentiation, spanning from the definitive endoderm to fully mature hepatocytes. For example, during the definitive endoderm phase, characteristic markers include sex-determining region Y-box 17 (*SOX17*) and forkhead box A2 (*FOXA2*) [119,120]. As cells progress to the hepatic specification stage, liver-specific transcription factors, plasma, and cytoskeletal proteins are expressed. For example, at this stage, hepatocyte nuclear factor 4 alpha (*HNF4a*), alpha-fetoprotein (*AFP*), albumin (*ALB*), and cytokeratin 18 (*CK18*) can be detected [121,122,123]. To distinguish mature hepatocytes, a combination of expression and functional analyses includes tryptophan-oxygenase, tyrosine amino-transferase, and specific cytochrome enzymes [123]. Nonetheless, evaluating gene and protein expressions alone is insufficient to fully characterize hepatocyte maturity. Hence, it is important to perform functional assays, including testing glycogen storage capacity, urea synthesis, albumin production, and enzyme activity [124,125]. These standard endpoints evaluate the efficiency of iPSC differentiation into hepatocytes. Thus, in this context, iPSC-derived hepatocytes offer significant advantages over many conventional in vitro liver models, particularly in terms of sample accessibility, differentiation potential, and the capability to produce patient-specific cells.

The differentiation of iPSCs to hepatocytes provides an abundant source of cells for studying genetic liver diseases and drug responses in a personalized manner, bypassing ethical dilemmas and lessening the potential for disease transmission that might arise when using hepatocytes sourced from human or animal donors. These hepatocytes can be used to study the development and maturation of liver cells, provide insights into the processes of liver organogenesis and regeneration, investigate disease mechanisms, and be utilized in drug discovery efforts to screen potential drug compounds for safety and efficacy.

### 4.4. Organoids Models Derived from Pluripotent Stem Cells

#### 4.4.1. Brain Organoids

Cortical brain organoids, also known as cerebral organoids, are three-dimensional (3D) cell culture models designed to replicate different aspects of the human cerebral cortex, which is responsible for intricate cognitive functions. The process of generating cerebral organoids typically involves patterning iPSCs into neuroectoderms using small molecules like those employed in neuronal fate patterning. Then, the neuroectoderm-induced cells self-assemble into spheroids, where spontaneous differentiation leads to the development of a physiological, laminar organization comprising neurons and glia. This neuroectodermal tissue is then embedded in droplets within a 3D matrix scaffold, which allows for studying spatial organization and gene expression patterns [126]. The 3D platform allows the incorporation of a diverse range of differentiated cell types found in living tissues, mimicking in vivo cell–cell and cell-matrix interactions [127,128]. Furthermore, these 3D culture conditions promote neuronal maturity and enhance neural and glial differentiation [129,130]. Brain organoids can then be maintained for extended periods in spinning bioreactors that enhance nutrient diffusion [127,128]. Upon maturation, morphological analysis has revealed cell specification markers for various brain regions, including forebrain, midbrain, and hindbrain, as well as sub-regional markers, while functional neural activity is evident by calcium oscillations, glutamate receptor activity, and axon branching. 

Brain organoid cultures can be scaled up for experimentation and used as a platform for drug screening, identifying potential therapeutic compounds, or evaluating the efficacy of drugs for various neurological conditions. They are essential for understanding and addressing a wide range of neurological disorders, including neurodevelopmental conditions such as autism spectrum disorders, neurodegenerative diseases like Alzheimer’s and Parkinson’s [131,132,133,134,135], as well as infectious diseases that affect the brain, such as the Zika virus [136]. This approach has the potential to reduce reliance on animal models and expedite drug development in the field of neuroscience. 

Brain organoids can be made into more complex structures, allowing us to investigate the interactions between different parts of the brain. This can be achieved by co-cultivating cortical organoids with organoids from different brain regions, such as the midbrain or hippocampus. This strategy incorporates relevant cell types to faithfully replicate the impact of non-cell-autonomous effects [134]. For example, co-culturing glial cells with neurons profoundly influences neuronal activity and the effectiveness of experimental therapeutics in vitro [137,138,139], as glial cells constitute a significant portion of human brain cells and play a crucial supportive role for neurons in both healthy and diseased conditions [140,141]. For example, brain organoids were used to investigate Giant Axonal Neuropathy (GAN), a neurodegenerative disease caused by mutations in the *KLHL16* gene. *KLHL16* encodes gigaxonin, a protein responsible for regulating the turnover of intermediate filament proteins. To study this condition, patient-specific GAN iPSCs and isogenic control cells, generated using CRISPR technology, were differentiated into brain organoids. These brain organoids exhibit pathogenic aggregates of neurofilament and GFAP proteins. Importantly, the GAN astrocytes within the brain organoids display a dense accumulation of intermediate filaments near the nucleus and abnormal nuclear morphology when compared to isogenic cells. This observed phenotype closely resembled the characteristics of the disease seen in patients [85].

The confinement of cells within specific regions of the 3D space can be accomplished through different methods, including bioprinting layers of hydrogels within microfluidic chips. These hydrogels have the capacity to replicate tissue architecture and facilitate precise cell–cell interactions, allowing for the development of assay systems to monitor neuronal migration and maturation phenotypes [142]. The versatility of bioprinting enables the adjustment of the composition and mechanical properties of layered matrices, resulting in a customizable platform capable of mimicking various developmental and disease states of brain tissue. Current bioprinting techniques are compatible with most standard matrix proteins, and novel matrices are being developed to enhance molecule delivery and finely control mechanical stress within 3D environments [143]. Screening platforms can be directly bio-printed in a Petri dish or utilized in conjunction with microfluidic principles to create highly intricate organ models that closely mimic in vivo environments [144,145]. Crucially, this technology also enables high-resolution imaging of human cell migratory behavior in a 3D context [142,146].

Brain organoids, while promising, do have their share of limitations, such as the absence of vascularization, restricted size, and variability in their development. This variability has hindered their automation and industrialization as primary screening platforms [136,147,148]. Considerable efforts are being directed towards improving the consistency and reproducibility of individual organoids through systematic optimization of culture media and growth conditions across different research laboratories [149]. Nevertheless, when compared to conventional cell cultures or animal models, brain organoids offer a more physiologically relevant and human-specific model. Importantly, it is crucial to acknowledge that iPSC-derived brain organoids raise ethical concerns. While these models lack the intricate complexity and consciousness of a fully developed human brain, they have ignited ethical debates regarding their level of consciousness and the necessity for ethical guidelines in research.

#### 4.4.2. Liver Organoids

Liver organoids generated from iPSCs have gained significant attention due to the liver’s crucial role in human health [150]. Similar to iPSC-derived neurons, the differentiation of iPSCs into hepatocytes mimics embryonic liver development [111]. Current techniques commonly use specific combinations of small molecules and different types of liver cells to enhance the efficiency of differentiation and replicate the complex hexagonal lobular structure. Efforts include combining liver cells such as hepatocytes, cholangiocytes, hepatic stellate cells, pericytes, Kupffer cells, liver sinusoidal endothelial cells, and portal fibroblasts [151]. Assessment of maturity and functionality is typically assessed by the expression of definitive endoderm and hepatocyte markers, as well as the demonstration of crucial liver functions like cytochrome P450 enzyme activity, glycogen synthesis, albumin secretion, and drug processing [111,112,113]. Thus, the resulting liver organoids composed of multiple cell lineages are able to naturally self-organize into iPSC-liver buds, mimicking phases of organogenesis, including the formation of a functional vascular-like endothelial network [152]. 

The integration of multiple cell types within a single structure presents a significant challenge. However, it promises to enhance organoid maturation and their capacity to perform various liver functions, such as glycogen synthesis, lipid accumulation, and metabolic activities. Indeed, transplantation of liver buds into a mouse model of acute liver failure has shown improved survival rates [153]. Thus, iPSC-derived liver organoid cultures exhibit enhanced maturation potential both functionally and morphologically. Accordingly, they represent a promising avenue for future research within the fields of liver biology and regenerative medicine [150,154].

#### 4.4.3. Brain–Liver Organoid Systems

In the human body, the brain and liver communicate through the blood–brain barrier (BBB) and the release of signaling molecules, affecting processes such as drug metabolism, toxicity, and overall homeostasis [155,156]. By co-culturing brain and liver organoids, we can study how the liver’s metabolic activities influence the response of neural cells. This system offers a unique platform for studying various aspects of physiology, metabolism, and drug responses, and it has the potential to advance our understanding of diseases that involve both the brain and the liver, such as SLC13A5.

A brain–liver co-culture system involves creating a controlled environment where brain and liver organoids (or cells) can interact and influence each other’s functions. For instance, brain and liver organoids can be physically close to each other in the same culture dish or cultured separately but share a common culture medium. Specialized microfluidic devices can enable controlled interactions between the two types of organoids while maintaining physical separation. However, the main challenge is having the appropriate culture conditions that support the viability and functionality of both types of organoids. Including the physiological barriers that exist between the brain and the liver, such as the BBB, would better represent the physiological environment [157]. Analyzing the interactions between the two organoids may involve measuring the release of metabolites, assessing gene expression, or using imaging techniques to visualize changes over time. This arrangement represents a complex and physiologically relevant model system to study how liver metabolism can influence neuronal function.

### 4.5. Challenges and Limitations of iPSC-Derived Model

Despite their great potential for disease modeling, cell therapy, and drug discovery, iPSC-derived cellular systems may have limitations and challenges that can affect reproducibility. For instance, reprogramming could introduce genetic alterations such as copy number variations, point mutations, or epigenetic changes, which might compromise the integrity of the iPSCs [158,159,160,161,162]. These alterations can introduce artifacts or confounding factors in subsequent studies using iPSC-derived progeny. Additionally, iPSCs inherently carry genetic and epigenetic imprints from their donor cells, contributing to variability and heterogeneity [160]. This can impact the cells’ ability to differentiate and their fundamental characteristics. In the case of SLC13A5 deficiency, mutations can influence the function and expression of the transporter differently. Therefore, iPSC-derived models may not consistently replicate the disease state in all patients due to these inherent variations and acquired changes during the reprogramming process.

The iPSC differentiation process into specific cell types can also be variable and inefficient, affecting the consistency and reproducibility of the experiments [98,99]. IPSCs often produce cells that resemble embryonic or fetal stages rather than adult ones [103,111,112,163]. This developmental discrepancy can influence the expression and function of genes and proteins and how cells react to drugs and environmental factors. For instance, iPSC-derived neurons and hepatocytes may not achieve the full maturity level of their counterparts in the human body. This is particularly relevant to SLC13A5 deficiency, primarily affecting the brain and liver—organs undergoing significant postnatal maturation and development.

Consequently, iPSC-derived neurons and hepatocytes might only partially mimic the disease phenotype and mechanisms observed in SLC13A5 patients. Overall, iPSC-derived models tend to have a limited scope for representing the complexity of diseases. These models are predominantly studied in an isolated, controlled in vitro environment, focusing mainly on cellular and molecular aspects [164]. This narrowed focus can overlook the intricate complexity of the whole organ system and the interplay among different cell types found in organisms. Therefore, these models might not fully capture the systemic manifestations of a disease or account for the influence of environmental factors on disease progression. This limitation highlights the need for cautious interpretation of findings and the importance of complementing them with other research methods, such as animal models or human tissue samples, to validate and refine findings for a comprehensive understanding of complex diseases such as SLC13A5. Finally, rigorous methods and standards are needed to generate, characterize, and compare iPSC models and apply appropriate controls and corrections to account for the variability and heterogeneity. Ongoing research and technological advancements continuously improve these models and address their limitations. Despite these challenges, iPSC-derived models are invaluable for understanding the pathophysiology of diseases like SLC13A5 and for developing targeted therapies.

## 5. CRISPR Genome Editing of Pluripotent Stem Cells

Induced pluripotent stem cells derived from patients with a genetic disease can be further advanced through the application of clustered regularly interspaced short palindromic repeats (CRISPR) genome editing technology. CRISPR is a genome editing tool used to precisely modify genes within cells [165]. This enables the introduction of patient genetic variants into a “healthy” background or the correction of pathogenic variants in patient-derived iPSCs [86,166,167]. In the context of introducing patient variants into a “healthy” background, iPSCs can be edited with CRISPR-Cas9 to recreate the genetic basis of the disease in a controlled laboratory setting. This allows for the generation of cellular models that mimic the exact genetic changes responsible for the disorder. For instance, the introduction of the pathogenic variant lysine 145 (K145) in TDP-43 (TAR DNA-binding protein 43) in iPSCs using CRISPR-Cas9 led to TDP-43 conformation changes. These changes impaired RNA-binding capacity and induced downstream misregulation of target genes in iPSC-derived cortical neurons. Expression of the variant in human iPSC-derived cortical neurons resulted in nuclear TDP-43 foci and loss of TDP-43 function [167]. In this context, genetically engineered iPSCs carrying specific *SLC13A5* variants found in patients can help better understand how specific mutations affect cell function. The neurons or hepatocytes derived from the SLC13A5-engineered iPSCs allow us to assess the impact of SLC13A5 dysfunction on cellular metabolism, neurotransmitter production, and overall neuronal/hepatocyte health and behavior. This approach facilitates a more precise understanding of the disease and supports the development of targeted therapies.

Similarly, isogenic controls are invaluable for conducting experiments where the impact of the specific mutation can be isolated and studied in comparison to healthy cells from the same individual to identify how the genetic changes influence cellular behavior. This can be particularly valuable for generating isogenic control lines, which are iPSCs derived from the same patient but with the corrected mutation. For example, using CRISPR-Cas9 to correct genetic variants causing Alexander disease (AxD), a fatal neurodegenerative disorder, helped unravel the disease mechanism. AxD is caused by mutations in glial fibrillary acidic protein (*GFAP*), which supports the structural integrity of astrocytes. AxD iPSC-astrocytes accumulated GFAP phosphorylated in Serine 13 (pSer13-GFAP) as aggregates within nuclear invaginations. This Ser13 phosphorylation promotes GFAP aggregation and targets it to proteolysis. Correction of the pathogenic mutation with the CRIPSR/Cas9 system in iPSCs abolished the Ser13 phosphorylation in iPSC-derived astrocytes and thus abolished the aggregates affecting the nuclear lamina [86]. Likewise, correcting patient mutations in the context of SLC13A5 disease can significantly enhance our understanding of the disorder. Editing specific mutations using CRISPR in patient-derived iPSCs allows for the observation of how these corrections affect cellular functions. This approach of direct comparison between cells with and without the mutation provides insights into the specific effects of the SLC13A5 mutation on cellular processes. Such studies can clarify the pathophysiology of the disease, and importantly, they can also aid in the development and testing of potential gene therapies or other treatments for precision medicine.

Finally, in the context of potential therapies, biomarkers can be used to evaluate the effectiveness of treatments by assessing if a particular therapy is having a positive or negative impact on the cellular level. Thus, CRISPR-Cas9 genome editing technology applied to iPSCs derived from SLC13A5 patients with genetic variants can help replicate the disease in vitro and enable the identification of disease-specific biomarkers. These biomarkers can have diagnostic, monitoring, and therapeutic implications. Thus, the integration of iPSC-based models with cutting-edge technologies such as CRISPR holds great potential for advancing our knowledge of both SLC13A5 neurological and liver disorders and for improving the lives of patients. 

## 6. Discussion

The precise pathophysiology underlying how SLC13A5 loss-of-function results in epilepsy refractory to treatment is a subject of open and ongoing research. Several hypotheses suggest SLC13A5 alters metabolic pathways, leading to neuronal dysfunction. Conversely, therapeutic inhibition of NaCT in the liver is a target to improve metabolic diseases, including non-alcoholic fatty liver disease, obesity, and insulin resistance. Thus, functionally accurate modeling and characterization of the mechanisms involved in citrate transport disruption are critical for understanding its role in human disease. 

**Figure 5:**
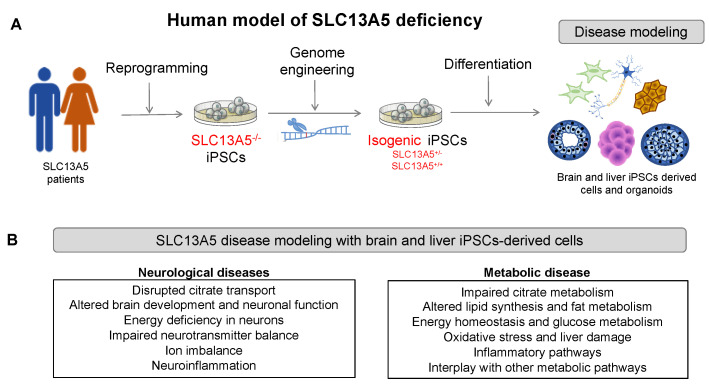
Proposed human model systems to study SLC13A5 deficiency. (**A**) Patient samples with SLC13A5 deficiency are reprogrammed to create induced pluripotent stem cells (iPSCs) that retain the genetic characteristics of the disease, which are crucial for understanding the genetics affecting disease manifestation. Isogenic control lines, genetically identical except for the disease specific mutation, can be created using CRISPR gene editing prior to their differentiation into brain and liver cells. (**B**) of SLC13A5 iPSC-derived brain and liver cells can be used to examine the neurological aspects and metabolic consequences, as well as their potential interactions.

IPSC-derived cellular systems are a powerful tool for modeling rare human genetic diseases, such as SLC13A5 (Figure 5). IPSCs derived from patients containing the genetic information of the disease can overcome the limitations of animal models, providing access to relevant human cell types that recapitulate the disease phenotype. For instance, patient-derived iPSCs differentiated into neurons or hepatocytes can be used to investigate molecular and cellular mechanisms, including citrate transport and accumulation, energy metabolism, oxidative stress, and other cellular processes. They can also be used to define the spectrum of the disease and how different mutations might lead to various disease severities, screen for potential therapeutic compounds that can restore the transporter function or ameliorate the symptoms, and enable personalized medicine approaches that can tailor treatments to individual patients based on their genetic background and disease severity.

Indeed, the use of iPSC-derived progeny holds great promise to advance our understanding of both the metabolic and epileptic components of SLC13A5 disease. Applying CRISPR genome editing technology to iPSC-derived patients with genetic variants can replicate the disease in vitro and enable the identification of disease-specific biomarkers. This allows for the generation of cellular models that mimic the exact genetic changes responsible for the disorder. For instance, iPSC-derived neurons from children affected by SLC13A5 mutations have the potential to faithfully replicate the physiological conditions specific to each patient, considering the genetic and epigenetic factors that may contribute to the epileptic phenotype. These iPSC-based models promise to clarify how neurons with *SLC13A5* variants respond to changes in citrate levels while elucidating the molecular and cellular mechanisms underpinning epilepsy and drug resistance in individual patients. 

The SLC13A5 iPSC-derived neurons, astrocytes, and brain organoids can inform us of the role of SLC13A5 in GABA synthesis and function. It is not fully understood how the depletion of intracellular citrate, resulting from SLC13A5 deficiency, affects the synthesis of neurotransmitters like glutamate and GABA in neurons. SLC13A5 transports citrate from the extracellular environment into the cell, where it serves as a precursor for the synthesis of acetyl-CoA, which is then used to synthetize GABA from glutamate [168]. GABA is an inhibitory neurotransmitter that regulates neuronal excitability [169,170]. Thus, a disruption in SLC13A5 function may affect acetyl-CoA availability for GABA synthesis [171]. Also, astrocytes play a role in maintaining neurotransmitter synthesis balance, participating in the glutamate-glutamine shuttle alongside neurons. In this shuttle, neurons release glutamate during neurotransmission, which astrocytes absorb and convert to glutamine, then send back to neurons for glutamate synthesis [172]. This cycle is vital for keeping glutamate levels in check, preventing excitotoxicity, and ensuring effective neurotransmission. Interestingly, low glutamate levels in the cerebrospinal fluid (CSF) of SLC13A5 patients [39] might impact the balance between excitatory and inhibitory neurotransmission, leading to epilepsy. Moreover, changes in acetyl-CoA levels can broadly affect neuronal function and GABA signaling [173]. Therefore, co-cultures of iPSC-derived neurons and astrocytes, or brain organoids, are valuable tools for investigating how SLC13A5 deficiency affects the glutamate-glutamine shuttle and to explore the NaCT’s role in citrate metabolism, neurotransmission balance, and GABA synthesis. 

SLC13A5 iPSC-derived cellular models can be utilized to study epilepsy caused by metabolic disturbances. For instance, neurons derived from iPSCs with SLC13A5 deficiency can reveal metabolic variations, potentially leading to energy deficits and neurotransmitter imbalances that heighten neuronal excitability. Metabolic imbalances can affect ion channel functioning and neurotransmitter systems, contributing to increased excitability [106,107,108]. Also, neurons rely on a continuous ATP supply to maintain membrane potential and ion gradients. A disruption in this balance can result in membrane depolarization. Additionally, imbalances in neurotransmitters may disrupt the brain’s excitation and inhibition balance, making neurons excessively excitable and prone to abnormal depolarizations. These speculations about SLC13A5 deficiency can be addressed using iPSC-derived progeny. 

A key question in SLC13A5 epilepsy is whether patients have an epileptic focus, the specific site where seizures originate, characterized by abnormal electrical activity. The varied nature of seizures observed in SLC13A5 patients [1,2,29], and the influence of the focus’s characteristics on seizure type and frequency suggest the possibility of an epileptic focus in these patients. Additionally, the abnormal firing of action potentials in neurons and the spread of hyperexcitability through interconnected brain cells could amplify seizure activity. As seizure activity typically ceases when inhibitory processes override excessive excitation, utilizing iPSC-derived neuronal systems alongside MEA technology could be instrumental in determining the excitability states induced by different *SLC13A5* variants. This approach could further our understanding of the presence and nature of epileptic foci in SLC13A5 epilepsy.

A critical question arising from SLC13A5 patients is whether SLC13A5 deficiency alters liver function. The relationship between SLC13A5 deficiency in both the liver and the brain revolves around the role of citrate transport and its function in metabolism, as the brain is highly dependent on a constant supply of energy, primarily in the form of glucose and other metabolites [174]. Thus, metabolic disturbances in the liver can indirectly affect the brain’s energy supply, potentially leading to alterations in neuronal function. Similarly, the liver plays a central role in regulating energy homeostasis in the body, while disruptions in hepatic metabolism, such as those caused by SLC13A5 deficiency, can lead to imbalances in both energy production and systemic utilization [39,175]. Hepatocytes import citrate through NaCT and use it as a substrate for acetyl-CoA, which is then used in the synthesis of fatty acids and cholesterol. Indeed, increased expression and activity of NaCT in the liver lead to elevated glucose production and increased synthesis of fatty acids and cholesterol. These changes promote insulin resistance, diabetes, obesity, and metabolic syndrome [39,171]. Thus, both loss and gain of function lead to metabolic disturbances that can have systemic effects, impacting various organs, including the brain [39]. The liver and brain are interconnected through metabolic pathways and signaling networks, making it important to investigate the crosstalk between these organs in the context of SLC13A5-related diseases.

## 7. Conclusions

The field of research using iPSCs in modeling SLC13A5 disease is coming of age, with several future directions holding promise to advance our understanding of both its metabolic and epileptic components. The combination of iPSCs derived from patients harboring *SLC13A5* variants and CRISPR-Cas9 technologies can help define the causative role of specific mutations and support the identification of biomarkers associated with SLC13A5 epilepsy. The models developed under such frameworks can delve deeper into defining any possible brain–liver relationship existing in patients with SLC13A5 deficiency. Going forward, these systems may enable biomarker identification and drug screening targeting the restoration of citrate transport and/or downstream pathways affected by SLC13A5 deficiency.

## Data Availability

Not applicable.
